# Supplementary material for: It was tough, but necessary. Organizational changes in a community based maternity care system during the first wave of the COVID-19 pandemic: A qualitative analysis in the Netherlands
Source: PLoS One. 2022 Mar 9;17(3):e0264311. doi: 10.1371/journal.pone.0264311 (PMC8906583; doi:10.1371/journal.pone.0264311)
Supplement: S1 Appendix — (DOCX) [file pone.0264311.s001.docx]

## Supporting information

**S1 Appendix. Topic list**

1. What influence did the corona crisis have on your work activities?
   - Adjustments in work activities
   - Changes in cooperation in the maternity care chain?
   - Stress
   - Influence on personal life
2. Which changes have/had a negative effect on the quality of maternity care and in which way?
   - Effects on medical outcomes
   - Effects on the experiences of women
   - Effects on maternity care providers (stress, lack of time, less income)
3. How did the process of making the changes in maternity care go during the COVID-19 crisis?
   - Your own role in this process

Who made the decisions?

- - Who and what influenced the decisions that were made?
  - Response speed to new developments or feedback
  - Communication in the field and to pregnant women

1. What is your opinion about the changes in maternity care that were made due to the corona crisis?
   - Proportionality
   - Timeliness; were the changes implemented in time and were they downscaled in time
   - Who were involved in drawing up the measures in your practice, organization or nationally?
2. Which innovations that were implemented could create opportunities for improving maternity care in the future?
   - Alternative ways to provide individual care
   - Innovative forms of organization

Opportunities to give the right care in the right place / substitution of care

- - Opportunities in the right type/quantity of care

1. Which challenges have caused you the most stress and why?
   - Job satisfaction
   - Degree of responsibility
   - Time pressure
   - Fear of infection
2. What effect did the COVID crisis and the associated measure have on cooperation within the maternity care?
   - Mutual relations
   - Information provision
   - Joint involvement in resolving problems in the maternity care chain
3. Are there other things you would like to say about this topic?
