# Supplementary material for: It was tough, but necessary. Organizational changes in a community based maternity care system during the first wave of the COVID-19 pandemic: A qualitative analysis in the Netherlands
Source: PLoS One. 2022 Mar 9;17(3):e0264311. doi: 10.1371/journal.pone.0264311 (PMC8906583; doi:10.1371/journal.pone.0264311)
Supplement: S2 Appendix — (DOCX) [file pone.0264311.s002.docx]

**S2 Appendix. Themes and subthemes**

**(Dis)proportionate measures**

- Changes in antenatal and postnatal care
- Downscaling outpatient care / Downscaling regular care
  - - Reduction in number of clinic visits and ultrasounds
- Keeping the number of contacts as low as possible
  - - Women need to attend clinic visits and ultrasounds by themselves
- Referral triage
- Less/no postpartum visitors on the maternity ward
- Changes in intrapartum care
- Labour
  - - Community-based midwives cannot assist women in hospitals
    - COVID-19 did not have a major influence on the maternity ward
- Separation of obstetrics department from the rest of the hospital
- Keeping the maternity care providers as much in their own department as possible
- Personal protection and hygiene measures
- Importance of protective measures
- Hygiene measures
- Proportionality of the measures
- Proportionality was good
- The measures were too strict
- Measures should have been initiated earlier
- Scaling up should have happened faster
- The measures are stricter in some regions than in others
  - - Discrepancy in impact of COVID-19 in different areas of the country
- Less downscaling, not so fast in a next pandemic

**A significant impact of COVID-19**

- Impact on maternity care providers
- Uncertainty
  - - Collective uncertainty
    - Uncertainty about COVID-19 as a disease in the beginning of the pandemic
- Effects on the mental state of maternity care providers
  - - Psychological burden
    - Overwhelming
    - Maternity care providers do not get the opportunity to restore their energy
    - Concerns about one’s own health / little concern about one’s own health (contrast)
    - Concerns about the wellbeing of others (colleagues?)
- Decrease in job satisfaction CONTRAST exciting new work activities
- Changes in on call schedule/ tasks
  - - More working from home
    - Increased organizational burden
    - Flexibility within the department
- Impact of the COVID-pandemic on work activities
- A lot of information in a short amount of time
- Effects on the relationship with women
- Less of a relationship with women
- Less attention to psychological wellbeing
- Perceived effects on the (expectant) mother and her partner
- More fear and uncertainty
- Only partner is allowed to be present during the birth
- New mothers could not visit their new-borns on the neonatology department if they had COVID symptoms
- Less partner involvement

**Differing views**

- Good cooperation within own echelon
- Good communication within the hospital
- Good ambiance in the hospital
- Great commitment among colleagues
- Use of a COVID-WhatsApp group
- Insufficient cooperation within own echelon
- Between different community midwifery practices
  - - Competition and friction between community midwifery practices
    - Different views and policy in different community midwifery practices
- Within department and hospital
  - - Differences (in policy) between different hospitals
- Good transmural cooperation
- Chain of command
- Taskforce/COVID-team
  - - Taskforce/COVID-team decides on measures
    - Care providers were well informed about measures that were instituted
- Clarity for care providers
- Coordination of policy with the policy of hospitals in the area
- Good cooperation within the maternity care chain
- Short lines within the maternity care chain
- Insufficient transmural cooperation
- Difficult regional communication
- Lack of clarity about upscaling
  - - The upscaling pace is different in different area’s
    - Community care scaled up too slowly
- Friction between community care providers and clinicians
  - - Community-based midwives refer women to the hospital too early
    - Risk selection shifted from community-based to hospital-based care
- Feeling of unity
- Enhanced team spirit

**Reluctance to seek care**

- Women experienced a higher threshold in contacting maternity care providers
- Avoidance of hospitals and community care setting because of women’s fear
- Women’s concerns and fears
- Women have withdrawn from care
- Patient delay
- Women experience a higher threshold to contact maternity care providers
- No increase in emergency referrals or complications despite a possible patient delay
- Women choose a different place to give birth
- More home births
- Only 1 person is allowed to be present during labour in the hospital

**Lessons learned**

- Increase in use of eHealth
- Increase in telephone and video visits
  - - Coaching through telephone
    - Benefits of eHealth for women
    - Downside of contacts by phone
      - Importance of psychical clinic visits
      - Benefits of face to face clinic visits
      - Importance of non-verbal communication
    - Differing opinions about follow-up by telephone
    - Preselect which visits can be done by telephone and which ones have to be face-to-face
- Other positive effects
- Less premature births
- Less postpartum visitors
- Women and maternity care providers experience the reduction in the amount of visitors as something positive
- Recommendations for the future
- Take a critical look at what care is really necessary
- Too many visits in maternity care / enough visits in maternity care (contrast)
- Take a critical look at what care is necessarily in hospital
- More customized care
- Centring COVID care in different hospitals
