# Supplementary material for: It was tough, but necessary. Organizational changes in a community based maternity care system during the first wave of the COVID-19 pandemic: A qualitative analysis in the Netherlands
Source: PLoS One. 2022 Mar 9;17(3):e0264311. doi: 10.1371/journal.pone.0264311 (PMC8906583; doi:10.1371/journal.pone.0264311)
Supplement: Attachment — Submitted filename: Review - It was tough but necessary PLOS.pdf [file pone.0264311.s003.pdf]

It was tough but necessary

- **Overall**: This is an interesting piece of research and very relevant in terms of how maternity care can better respond to unprecedented health crisis as well for what can be learned from the COVID-19 response. There is a need to reform practices and build preparedness for future possibilities of crisis like the current pandemic. It is also relevant in presenting the perspectives and voices of frontline healthcare providers, often missed in policy formulations and recommendations.
- **Title**: The title reads 'It was tough but necessary' , obviously taking from what emerged as their overarching theme. However, in reading the findings, it seems there are varied opinions that suggest that not everybody felt the measures were necessary. For example, the community-based midwives tend to have a different opinion on not being allowed to visit even when the clients did not have symptoms or the fact that in the north, the respondents did not agree with the timing or felt the measures were too drastic. You may revisit your title
- **Methods:**
    - The authors mention the word 'reflexivity' but do not demonstrate how this was applied and how it may be reflected in study findings, except to say who the researchers were.
- **Sampling**
    - They do not mention what sampling strategy was used and how did they arrive at their sample size
    - Although the authors provide a link - it would be useful if they provide a sense of the sample of the mix-methods study - so that one could locate this study in the larger context and get a sense of how the qualitative sample matches up to the overall sample.
    - They state that the sample was representative. What is not clear is what was the overall universe for this to be representative of The paper suggests that all hospitals and community-based midwifery practices are uniform across the country. It may be so. However, if it is not, then the authors may want to provide some sense of where the providers are located, as clearly positive and negative perceptions on the measures would vary upon the type of facility and the context.
    - In the study design the authors mention – "that they wanted to 'weigh' the measures taken against the COVID-19 pandemic, to establish if there was balance between the measures taken and the impact that they had". This is a qualitative study - how do they weigh the measure and that too without having a real sense of the impact – perhaps they could consider rephrasing.

- **Need for a context**: The paper will gain immensely if the authors could provide a brief context section that gives the broad outline of the Dutch health system (that they mention as 'unique' towards the end but don't say how or why); what was the exposure to the pandemic in Netherlands and what was the broad response strategy of the health system; and how it affected normal functioning. This will help the findings in the context – for example, why were the changes in the maternity care protocol made? It could also help explain some of the quotes where the hospital midwifes state that they had very high burden (line 238). Also, in a quote it states that it escalated fast, the number of cases kept

increasing etc. While this is in a quote, but we have no background to interpret the quote against.

- **Findings and Analysis**
  - Given that the sample comprises 3 types of respondents, all of whom vary either in terms of their specialisation, position and/or location, it would have been useful to locate their responses in specific contexts. The authors mention several times that community-based midwifes had a different experience in terms of the restrictions on meeting in-person, or the non-uniform implementation of the national guidelines etc. However, they don't state why this was the case - how did their specific practice of being community-based midwifes vary from that of those who were hospital-based and how much of their perception is related to the specificities of their location and practice.
  - There are some unsubstantiated statements made in specific sections. For instance, in line 183 they mention the word 'fear' and concern and the quote talks about 'pity' and concern – which is not the same as fear. Similarly, they mention that this 'negatively impacted their wellbeing' (line 183-184), but they don't say how? In the absence any other data, how is this to be established?
  - The authors may want to rephrase what appear to be inconsistencies. In line 189-190 they say impact of COVID-19 was not noticeable in the maternity wards but later they mention that respondents talked of the escalating numbers and work burden
  - In line 194-195, the authors talk of the impact on outpatient delivery and intrapartum care, but in the absence of an overall context it is not clear why it was done, what the scale of this was and thus to know what the potential impact of this might be. If the responses could be reflected against what the national scenario was on these issues, the significance of this may be clearer.
  - Similar inconsistency is seen in line 204-206. "There was a difference of opinion between maternity care providers about the proportionality and timeliness of the measures that were implemented. Overall, most participants were content with the proportionality and timeliness of the measures". The two sentences seem do not reconcile. There are different categories of respondents and different locations – could these differences arise because the category they belong to? If so, can we club them together when we say, 'overall most participants were content'? also the text that follows does not seem to be consistent with this interpretation.
  - In line 217-219 quote, not much is said about the awareness levels and backgrounds of the midwives that may have informed their opinions. For instance, here the statement indicates that she may not be aware that people can be infected and yet be asymptomatic – if aware, it may have lead the respondent to assume that the restriction was justified?
  - In line 224, without adequate background and context it is difficult to understand terms like "capacity problem".
  - The authors talk about cooperation and communication as a separate section (291) but they also provide details about cooperation in the hospitals in the previous section (258-organisational effect). They may want to club them together
  - Overall, I feel that the findings under each section needs to be structured for the reader to be able to better differentiate the varying perceptions among the different

categories /regions and why? In the way it is currently organised, these differences seem embedded in the flow of the narrative and do not clearly standout.
  - We are dealing with different types of care providers - how did their different professional positions affect the differences of opinions between them? For example, when you say some maternity providers are not on the same page when it comes to certain policies (in the section on coordination and communication)
  - Line 299-300 "There are, however, notable differences between the experiences of obstetricians, residents and hospital-based midwives, and the experiences of community-based midwives." This comes much later but I felt that this frame needs to be set up early in the findings sections and all the findings reflected along these lines
  - In line 323-324, if the task force was seen effective by all then why did the community-based midwifes not communicate that there was variation in midwifery practices contravening the national guideline. This the authors mention created competition and rift. There is some inconsistency here that needs to be addressed.
- **Discussions**
  - The discussion section reads well and is nicely done.
  - It discusses the pros and cons of the key issues that have emerged but does not really suggest the 'then what' or 'so what?' For example, one size does not fit all, and gradation would lead women to shop. What are some of the potential ways in which this issue could be addressed or what policy implication might this have?
  - In their discussion the authors may want to refer to examples in other countries where COVID hospitals have been set up, especially on their feasibility. Also, their conclusion on inadequate availability of staff seems to suggest that staff from maternity care were also on COVID duty. Some clarity here would be good.
  - The findings lend themselves to the medico-techno discussions on pre-mature birth, which also feels incomplete given the number of variables affecting pre-mature birth.
  - Given that the authors look at the perception of the providers – the discussions on personal experience and challenges have not been adequately covered – given that so much has been written on health worker challenges in COVID-19, what kinds of stresses did they face outside of just being scared of the infection? Why did they have to do COVID work? They talk of the 'burden', but how did that affect them emotionally and physically and how did it in turn impact their work?
  - There are some aspects that emerge only in the discussion e.g. PPEs for provider, Dutch health systems being unique etc, without it being reflected in the findings.
  - I felt that differences in the different cadre or 'echelon' has to be discussed with some context as well as policy implications. The findings suggest that not only did the community-based midwives experience the measure differently from the hospital-based providers, but the difference between them seem to have a direct bearing on care provision (excess referrals that increased workload in the hospital). You mention that the existing differences was aggravated by COVID-19. Some elaboration on this would help.
