# Supplementary material for: It was tough, but necessary. Organizational changes in a community based maternity care system during the first wave of the COVID-19 pandemic: A qualitative analysis in the Netherlands
Source: PLoS One. 2022 Mar 9;17(3):e0264311. doi: 10.1371/journal.pone.0264311 (PMC8906583; doi:10.1371/journal.pone.0264311)
Supplement: Attachment — Submitted filename: Response to Reviewers.docx [file pone.0264311.s004.docx]

Department of Obstetrics & Gynaecology	Department of Midwifery Science

Amalia Children’s Hospital	AVAG/ Amsterdam Public Health research institute

Radboud University Medical Centre	VU University Medical Centre

Geert Grooteplein 10, 6525 GA Nijmegen	Van der Boechorststraat 7, 1081 BT Amsterdam

The Netherlands	The Netherlands

*30 July 2021,*

Dear editors,

We thank the reviewers for their time and effort to critically revise our paper and we believe that this has helped to improve our article . In this letter we respond to the comments one by one. We copied the reviewer’s comments in Italic, and responded to them below.

First reviewer’s comments:

*'On its arrival' sounds as if COVID came for a holiday, and detracts from the gravity of the virus - perhaps watch your language here to ensure academic style is maintained throughout.’*

We changed ‘on its arrival in the Netherlands…’ to ‘When the number of COVID-19 cases in the Netherlands increased excessively...’

*‘Not sure what you mean by 'affinity with maternity care'.’*

With this sentence we mean that the authors of this manuscript are all either working in maternity care in different functions, for example as a registered midwife or gynaecologist, or are in another way involved in maternity care in the Netherlands.

*‘You may have to clarify what you mean by 'residents' - as this will have different meanings depending on the country. For example, whilst it is occasionally use in the UK for junior medical staff, the more common use of residents is as a synonym for inhabitants or occupants.’*

We were under the impression that ‘resident’ is a fairly well known term for trainee medical

specialist, especially in the US. We have changed residents into ‘obstetrical residents’.

*‘Is you sampling plan table representative of the actual participants or who you hoped to interview. This needs to be clarified.’*

As stated in the article, we purposely sampled respondents from different regions and

different professions. The sample covers all of the participants. There were others who

responded to our call but were not  interviewed. This was because they were similar in their location of residence and work situation to those we had already included.

*‘Your data analysis section is where I have an issue. You appear to have what I would deem, 'methodological pluralism' whereby you are drawing upon a mixture of analytical methodologies to explain what you may have actually done. You suggest you are using a constructivist approach to thematic analysis, but you cite methodology from Charmazian Grounded Theory and discuss open, selective, and axial coding which are all reminiscent of Grounded Theory. I would suggest you either align your methodology to Grounded Theory (if you are wanting to develop a theory from your data) OR align your methodology to Thematic Analysis (if you are wishing to identify the broad base themes present in your data). I would also avoid use of terminology such as code-tree which makes it sound as if you have used a codebook, which again would not be appropriate or relevant for Grounded Theory or Thematic Analysis.’*

Our approach was consistent with Thematic Analysis. We did indeed wish to identify the broad themes from our data. We have adjusted the segment in the article accordingly in Line 183-191.

‘In our study, a Thematic Analysis was used to analyse the interviews[19]. Qualitative data analysis software program Atlas.ti was used for coding[20]. The coding process was started with reading through all the transcripts carefully to gain a deep understanding of the content. Next, inductive coding was performed. New codes were added as new interviews were analysed. The codes were grouped into subthemes and merged into themes by IA and MH (S2 Appendix). MH coded one interview at the beginning of the analysis and ST coded two interviews halfway through the process to check for any missing codes. Finally, the themes were tested on all the interviews by LB. Differences of opinion regarding the themes were resolved through discussion between the authors.’

*‘Please could you explain the reasoning as to why ethical approval was deemed not necessary.’*

The ethical committee of the University of Amsterdam deemed this study as not needing

ethical approval for the following reasons: there were no patients involved, there were no

medical procedures performed on the participants, and participation in the study was not expected to cost the participants an undue amount of time. In addition, participation was not deemed to be too emotionally challenging for the participants.

*‘From reading your results, it appears you could equally write this up as a Thematic Analysis OR a Grounded Theory. Given the steps of coding you have done, and the fact that you appear to have an over-arching theory at the end of your results section (rather than an overarching theme as you have called it, or Central Organising Concept as it would be called in Thematic Analysis), I would recommend you amend your manuscript to describe a Grounded Theory analysis rather than a Thematic Analysis. I would then spend some more time describing what you have called an 'overarching theme', but describe it as an Overarching Theory instead (but you would not present data or quotations under the Overarching Theory, so please remove this quotation). This would then echo your title, which makes it instantly look like a Grounded Theory, as you have the Theory in the title already (which made me first think this was a Grounded Theory paper). Your work would be much better suited to a Grounded Theory and I think you would have a stronger manuscript for it if you made these changes. Seek further advice from Silverio et al., 2019 - (Re)discovering grounded theory for cross-disciplinary qualitative health research; for the steps required and terminology used in Grounded Theory that you will need to imbed into your paper, and systematically remove all reference to thematic analysis throughout the paper.’*

As stated above, we feel that our approach has been more in line with Thematic Analysis, as we wished to identify broad themes from our data. We adjusted the analysis to a Thematic Analysis, and described the former Overarching Theme as a Central Organising Concept now, which can be read in Line 183-191.

‘*What type of data saturation did you use? On what principles did you judge data saturation? Francis et al., 2010 - what is an adequate sample size OR Guest et al., 2006 - How many interviews are enough OR Malterud et al., 2015 - Sample size for interviews OR Saunders et al., 2018 - Saturation in qualitative research OR Vasileiou et al., 2018 - Interviews study sample size - would all be good, but different ways of explaining data saturation - so it would be good to know where you positioned yourself.’*

We decided data saturation was reached when after coding four consecutive interviews, no new codes had emerged. Our final number of 19 respondents is significantly higher than the number of 6-12 suggested by Guest in the paper the reviewer cited, which seems to be the average for obtaining the main themes in qualitative health research.

*‘Your theme names require a bit of interpretive refinement. At the moment they are very descriptive and they need to be worked up a little more to be a bit more refined and interpretive. They should also be memorable rather than basic descriptions. They need a bit more analytic work.’*

We adjusted the theme names to the following:

‘Measures that changed maternity care and their proportionality’ to ‘(Dis)proportionate measures’,

‘The impact of COVID-19 was significant’ to ‘A significant impact of COVID-19’,

‘Different views on communication and cooperation’ to ‘Differing views’,

‘Threshold for women to seek care’ to ‘Reluctance to seek help’

and ‘Some things were not so bad’ to ‘Lessons learned’.

*‘When you state 'over-arching theme' do you mean this as a Central Organising Concept (in the case of Thematic Analysis) or do you mean this as a Theory (in the case of Grounded Theory). Here it is very unclear as to what analytic methodology you have used and unfortunately, this severely weakens your paper.’*

We indeed mean to describe a Central Organising Concept. We made adjustments to this part in Line 183-191.

*‘It is entirely unclear what you mean by 'divided into two themes'. Which themes are you now talking about?’*

We changed this sentence into: ‘divided into two domains’, to distinguish this from the

themes of the study itself.

*‘It is a bit long and could be tightened/sharpened to ensure you keep hold of the reader's interest.’ (This comment is about the discussion section).*

We have tightened the discussion section where needed (Line 493-603).

*‘I am unsure as to why you have so strongly focused on preterm birth in your discussion, which was not overly evident throughout the rest of your paper - possibly consider toning down?’*

We took your advice and adjusted this section, by including some more literature.

*‘I think you have underplayed your strengths. The fact you did this in the early phases of the pandemic to give us a snapshot of the Dutch context; the fact you have used a rigorous methodology (although you have not written it up exact enough as yet); the diversity of your participants.’*

We have added some information in Line 606-613.

**‘**There are several strengths to this study. The diverse nature of the study group and research team and recruitment from all parts of the country enabled the capture of a wide variety of opinions in this study. Another strength is the diversity of our participant group, which created a representative sample of maternity care providers in the Netherlands. In addition, this study was conceived and initiated very early on in the pandemic, with interviews taking place at the end of the first wave. This provides a unique insight into the opinions of Dutch maternity care providers in the midst of restriction measures. Finally, we used a rigorous methodology, underpinning the validity of our findings.’

*‘I would disagree with your point about a pre-determined topic list not being a major issue, especially as you detail above that other questions were introduced into interviews if they arose, thus meaning your topic guide was not suitable enough. It's okay to have limitations - we all do - just be a little more honest about them.’*

In fact, this limitation was about the potential bias because some participants knew the interviewers and the interviewers were an obstetrician and midwife themselves. We have adjusted the text to make this clear in Line 612-621.

‘There are, however, some limitations as well. It may be that these findings are not applicable to other countries and healthcare systems, because of the uniqueness of Dutch maternity care. However, the measures that the participants outlined in this study, have also been described in other countries[21]. Another possible limitation is that some of the participants were known to the interviewers through personal networks. This may have coloured their comments to a certain extent. In addition, participants might have given socially desirable answers because the interviewers were a midwife and gynaecologist themselves. Considering the variety of opinions that were expressed, this does not appear to have been a major problem.

Second reviewer’s comments:

*‘The title reads ‘It was tough but necessary’ , obviously taking from what emerged as their overarching theme. However, in reading the findings, it seems there are varied opinions that suggest that not everybody felt the measures were necessary. For example, the community-based midwives tend to have a different opinion on not being allowed to visit even when the clients did not have symptoms or the fact that in the north, the respondents did not agree with the timing or felt the measures were too drastic. You may revisit your title.’*

After careful consideration with all the authors, we decided to not change the title, as this title does represent the overall theme of the study.

*‘Need for a context: The paper will gain immensely if the authors could provide a brief context section that gives the broad outline of the Dutch health system (that they mention as ‘unique’ towards the end but don’t say how or why); what was the exposure to the pandemic in Netherlands and what was the broad response strategy of the health system; and how it affected normal functioning. This will help the findings in the context – for example, why were the changes in the maternity care protocol made? It could also help explain some of the quotes where the hospital midwifes state that they had very high burden (line 238). Also, in a quote it states that it escalated fast, the number of cases kept increasing etc. While this is in a quote, but we have no background to interpret the quote against.’*

We apologise if this was unclear to the reviewer. It is not the Dutch healthcare system that

is unique, but the Dutch maternity care system, with its independent primary care midwives

and relatively high percentage of homebirths. This has been clarified in the introduction section. We have added some background on exposure to the pandemic and the initial response (Line 74-100).

*‘The authors mention the word ‘reflexivity’ but do not demonstrate how this was applied and how it may be reflected in study findings, except to say who the researchers were.’*

We have added some sentences to clarify this in Line 130-142.

**‘**The interview team consisted of a medical student (IA), an obstetrician (MH) and a midwife (ST). The interviews with the obstetricians and obstetrical residents were conducted by MH, and the interviews with the midwives by ST. During the interviews, IA was present to record the conversations. All of the authors have an affinity with maternity care, and are working in maternity care in different functions, for example as a registered midwife or gynaecologist, or are in another way involved in maternity care in the Netherlands. …’

*‘In the study design the authors mention – “that they wanted to ‘weigh’ the measures taken against the COVID-19 pandemic, to establish if there was balance between the measures taken and the impact that they had”. This is a qualitative study - how do they weigh the measure and that too without having a real sense of the impact – perhaps they could consider rephrasing.’*

We have adjusted the text based on the reviewer’s comments in Line 144-150.

‘This study is part of a larger multidisciplinary study, the WAAG -study. Historically, in the Netherlands, each municipality had a ‘waag’, a communal weighing scale, used for measuring goods. In this context, we wanted to examine professionals’ experiences with the changes in the organisation of maternity care which were aimed at balancing (‘weighing’) providing good quality care versus limiting the spread of COVID-19.’

*‘Although the authors provide a link - it would be useful if they provide a sense of the sample of the mix-methods study - so that one could locate this study in the larger context and get a sense of how the qualitative sample matches up to the overall sample.’*

We refer to our reply to the comments of reviewer 1. This was a purposive sample,

representative of all parts of the country and different settings and types of professionals.

The quantitative study is referred to above.

*‘They state that the sample was representative. What is not clear is what was the overall universe for this to be representative of The paper suggests that all hospitals and community-based midwifery practices are uniform across the country. It may be so. However, if it is not, then the authors may want to provide some sense of where the providers are located, as clearly positive and negative perceptions on the measures would vary upon the type of facility and the context.’*

There are 10 perinatal (tertiary) centres, and 71 regional hospitals and about 500 midwifery practices throughout the country. All women have access to a midwifery practice, regional hospital or perinatal centre, depending on their ned. In the case of the first wave, there were regional differences caused by the severity of the outbreak being centred in the south, hence the purposive sampling based on region of residence. We have added information on the region of the maternity care professional for each quote.

*‘They do not mention what sampling strategy was used and how did they arrive at their sample size.’*

See earlier comments and replies to this reviewer and reviewer 1. The sample size was

determined by both data saturation and the expected minimum of participants needed to

have input from each of the different settings.

*‘Given that the sample comprises 3 types of respondents, all of whom vary either in terms of their specialisation, position and/or location, it would have been useful to locate their responses in specific contexts. The authors mention several times that community-based midwifes had a different experience in terms of the restrictions on meeting in-person, or the non-uniform implementation of the national guidelines etc. However, they don’t state why this was the case - how did their specific practice of being community-based midwifes vary from that of those who were hospital-based and how much of their perception is related to the specificities of their location and practice.’*

Part of the answer is explained in the section on the Dutch maternity care system. Community based midwives work independently, and with low risk women. Hospital based midwives are part of the medical team working under the supervision of obstetricians, together with obstetric residents. They see the referrals from the community based midwives, in case a woman becomes high risk. This has been added in the introduction, in Line 62-73. In addition, we have added the location of the respondents to their quotes.

*‘Overall, I feel that the findings under each section needs to be structured for the reader to be able to better differentiate the varying perceptions among the different categories /regions and why? In the way it is currently organised, these differences seem embedded in the flow of the narrative and do not clearly standout.’*

In our study, we wanted to obtain a representative and varied sample to make sure that we could show all the different experiences. Our purpose was not to compare different groups to each other. Only where the differences were very remarkable and the experiences had something to do with the setting in which the maternity care providers worked, we enlightened that in the manuscript. Besides that, we have added more background about the respondents with each quote.

*‘There are some unsubstantiated statements made in specific sections. For instance, in line 183 they mention the word ‘fear’ and concern and the quote talks about ‘pity’ and concern – which is not the same as fear. Similarly, they mention that this ‘negatively impacted their wellbeing’ (line 183-184), but they don’t say how? In the absence any other data, how is this to be established?’*

We have adjusted our text according to this comment in Line 219-222.

‘Many participants believed that women did not like these measures these measures increased fear and concern among pregnant women and, therefore, negatively impacted their wellbeing. since they missed some elements of care they would have liked to have had.’

*‘The authors may want to rephrase what appear to be inconsistencies. In line 189-190 they say impact of COVID-19 was not noticeable in the maternity wards but later they mention that respondents talked of the escalating numbers and work burden.’*

In line 189-190, we indeed state that the impact of COVID-19 was not very noticeable on the maternity ward. What we mean by that is that there we not many COVID-19 infected patients on the maternity wards. However, the work burden was mainly increased because of sickness of maternity care workers which created a higher work burden for the maternity care providers that were not infected with COVID-19. Also, some maternity care workers indicate that the higher burden came from making protocols for COVID-19 care. So the higher burden did not came from taking care of COVID 19 infected patients. We have added some text to

clarify this in Line 276-286.

**‘**Some participants stated that there was such a high burden on them, that they did not have time to recharge their energy. The work burden was mainly increased because of sickness or mandatory self-isolation of maternity care workers, which created a higher work burden for the others. Also, some maternity care workers indicate that the higher burden came from making protocols for COVID-19 care.’

*‘In line 194-195, the authors talk of the impact on outpatient delivery and intrapartum care, but in the absence of an overall context it is not clear why it was done, what the scale of this was and thus to know what the potential impact of this might be. If the responses could be reflected against what the national scenario was on these issues, the significance of this may be clearer.’*

We have made some adjustments in the introduction section in Line 74-100.

*‘Similar inconsistency is seen in line 204-206. “There was a difference of opinion between maternity care providers about the proportionality and timeliness of the measures that were implemented. Overall, most participants were content with the proportionality and timeliness of the measures”. The two sentences seem do not reconcile. There are different categories of respondents and different locations – could these differences arise because the category they belong to? If so, can we club them together when we say, ‘overall most participants were content’? also the text that follows does not seem to be consistent with this interpretation.’*

We have made some adjustments in this section in Line 245.

*‘In line 217-219 quote, not much is said about the awareness levels and backgrounds of the midwives that may have informed their opinions. For instance, here the statement indicates that she may not be aware that people can be infected and yet be asymptomatic – if aware, it may have lead the respondent to assume that the restriction was justified?’*

We have added clarification in Line 254-255:

‘At the time of the interviews, the official position of the Dutch government was still that there was no asymptomatic spread of the virus.’

*‘In line 224, without adequate background and context it is difficult to understand terms like “capacity problem”.’*

We have amended the text to clarify in Line 263-267.

*‘We are dealing with different types of care providers - how did their different professional positions affect the differences of opinions between them? For example, when you say some maternity providers are not on the same page when it comes to certain policies (in the section on coordination and communication.’*

We have added some text to the introduction to clarify in Line 60-100.

*‘The authors talk about cooperation and communication as a separate section (291) but they also provide details about cooperation in the hospitals in the previous section (258-organisational effect). They may want to club them together.’*

We feel the organizational changes described in the previous segment are different from

the (interdisciplinary) cooperation described later in the next theme. We have therefore

chosen to leave them where they are.

*‘Line 299-300 “There are, however, notable differences between the experiences of obstetricians, residents and hospital-based midwives, and the experiences of community-based midwives.” This comes much later but I felt that this frame needs to be set up early in the findings sections and all the findings reflected along these lines.’*

We did consider this strategy early on in the writing of this article. However, we decided

against it because it would put too much emphasis on the differences in experiences

between the two echelons. That was not the main component of our study, and certainly not

a major finding. We have therefore decided to mention this difference when needed, but to not overemphasize it.

*‘In line 323-324, if the task force was seen effective by all then why did the community-based midwifes not communicate that there was variation in midwifery practices contravening the national guideline. This the authors mention created competition and rift. There is some inconsistency here that needs to be addressed.’*

This section is about transmural communication, meaning between echelons (hospital and

primary care). This is something the respondents were unanimously positive about. They are

referring to everybody being on the same page about whatever new agreements and

guidelines were communicated that day. Or sometimes several times per day. Those were appreciated, even though some practices may not always have adhered to everything.

*‘It discusses the pros and cons of the key issues that have emerged but does not really suggest the ‘then what’ or ‘so what?’ For example, one size does not fit all, and gradation would lead women to shop. What are some of the potential ways in which this issue could be addressed or what policy implication might this have?’*

We do make suggestions for then wat, such as the ‘tier’ approach as in the UK. In Line 509-512, and we have added some other suggestions, for example in Line 267-269.

*‘In their discussion the authors may want to refer to examples in other countries where COVID hospitals have been set up, especially on their feasibility. Also, their conclusion on inadequate availability of staff seems to suggest that staff from maternity care were also on COVID duty. Some clarity here would be good.’*

In fact, no staff were on COVID duty. Staff was short because people were off sick, or in quarantine because of contact with a sick patient or family member. Some changes were made to clarify this. References to COVID hospitals were added.

*‘Given that the authors look at the perception of the providers – the discussions on personal experience and challenges have not been adequately covered – given that so much has been written on health worker challenges in COVID-19, what kinds of stresses did they face outside of just being scared of the infection? Why did they have to do COVID work? They talk of the ‘burden’, but how did that affect them emotionally and physically and how did it in turn impact their work?’*

The purpose of this study was to study the effect of the pandemic on the organisation of maternity care through the eyes of the providers; our purpose was not to study how health care staff was affected by working on COVID wards. None of our respondents worked on a COVID ward. And nationwide, very few did. Some nurses and residents did, but no obstetricians or midwives, since they could not be missed in maternity care. And their experiences on COVID wards, although interesting, would have fallen outside the scope of this study. The ‘burden’ mentioned is the hard work, the stresses of the time, and being short staffed due to colleagues being absent, as described above. This has been added at certain places in the manuscript.

*‘The findings lend themselves to the medico-techno discussions on pre-mature birth, which also feels incomplete given the number of variables affecting pre-mature birth.’*

We agree, therefore we have shortened this section, and refer to a recently published Dutch paper that provides more detail on this subject in Line 560-573.

*‘The authors may want to highlight more effectively the main contributions that the paper is making.’*

We have added this information on strengths and weaknesses in the discussion section in Line 608-624.
