# Supplementary material for: It was tough, but necessary. Organizational changes in a community based maternity care system during the first wave of the COVID-19 pandemic: A qualitative analysis in the Netherlands
Source: PLoS One. 2022 Mar 9;17(3):e0264311. doi: 10.1371/journal.pone.0264311 (PMC8906583; doi:10.1371/journal.pone.0264311)
Supplement: Attachment — Submitted filename: Rebuttal letter.docx [file pone.0264311.s005.docx]

Department of Obstetrics & Gynaecology	Department of Midwifery Science

Amalia Children’s Hospital	AVAG/ Amsterdam Public Health research institute

Radboud University Medical Centre	VU University Medical Centre

Geert Grooteplein 10, 6525 GA Nijmegen	Van der Boechorststraat 7, 1081 BT Amsterdam

The Netherlands	The Netherlands

*7 December 2021,*

Dear editors,

We thank the reviewers for their time and effort to critically revise our paper and we believe that this has helped to improve our article. In this letter we respond to the comments one by one. We copied the reviewer’s comments in Italic, and responded to them below.

First reviewer’s comments:

*‘Overall, I am happy with the revised manuscript, but I have two outstanding issues - firstly I would still counsel you against presenting data under the central organising concept, and secondly where you have stated 'obstetrical resident', I think this can be renamed 'obstetric resident'.’*

Thank you for your positive remarks! We believe your previous comments have improved the manuscript significantly. In response, we have changed the term ‘obstetrical resident’ to ‘obstetric resident’.

Regarding the term Central Organising Concept: at first we used the term *overarching theme.* This was rejected by both reviewers in favour of *central organising concept*, because they deemed the study used thematic analysis rather than grounded theory. We agreed, and changed both the term and the methodological description. We do, however, feel that we can illustrate the meaning of our COC by one single all-encompassing quote. We have done the same in previous published work by this group (see Hollander et al. BMC Pregnancy and Childbirth 2017, Hollander et al. POLS One 2020 etc.), even though those studies did use grounded theory.

Second reviewer’s comments:

*‘I still feel that the piece could be sharper and more tightened – some data repeats itself (like women were not allowed to be accompanied by their husband or doulas).’*

We agree there are some semi-overlapping sentences. However, this is unavoidable in studies of this type. The results are delivered as findings without interpretation. The interpretation follows in the discussion, with a short recap of the results to illustrate the issue.
We have already almost cut the discussion in half during the first round of feedback and improvements, and in our opinion reducing it further will not do justice to the whole corpus of data.

*Some of the sub-themes are inconsistent and does not say much like ‘differing view’ while one clearly states the subject of the specific content – reluctance to seek care.’*

We agree that *differing views* can be interpreted in many different ways. We have changed the term to ‘*differing views on inter-provider cooperation’.* We believe this clarifies the content of this section better.

*‘I am still not sure what the unique contribution of the study was – what is that this study helps highlight that is not available from the larger study – the only uniqueness to me is the timing of the study.’*

The larger study consists of several sub-studies, of which this is one. We believe that, in order to understand a situation as unique as the first wave of the COVID-19 pandemic in depth, both quantitative and qualitative data are needed. The quantitative data provided insight into the scale of the problem, and may suggest causes and effects (this study has already been published, see van Manen et al. PLOS One, 2021). However, qualitative analysis is needed to answer questions as to why certain things occurred/were done. These two research strategies complement each other, and the total is worth more than the separate studies. In addition, we believed surveying both clients and care providers would provide a 360 degree overview on this confusing and intense time (especially for pregnant women). The studies involving qualitative and quantitative data on client experiences are currently in progress.

We have incorporated this rationale in our introduction and have moved some segments from methods to introduction (Line 117-132).

‘To date, several studies have investigated experiences of maternity care providers’ experiences with specific changes in maternity during the COVID-19 pandemic, such as the increase of e-Health [11,12]. Furthermore, several studies show that care providers' emotional health was under pressure[13,14]. However, as far as we know there have not been any studies who investigate the overall experiences of maternity care providers with the changes in maternity care during the first wave of the COVID-19 pandemic. In order to understand the experiences of maternity care providers during a situation as unique as the first wave of the COVID-19 pandemic in depth, both quantitative and qualitative data are needed. The quantitative data provided insight in the scale of the problem, and may suggest causes and effects, as can be seen in this recently published study [15], however, qualitative analysis is needed to answer questions as to why certain things occurred/were done. In this current study, we aimed to investigate which policy changes in maternity care during the first COVID-19 wave were perceived as positive by care providers and could offer opportunities for future improvements. In addition, we also wanted to know which changes were perceived as unfavourable by care providers and therefore needed re-evaluation in case of another crisis. Finally, we were interested in evaluating cooperation between different maternity care providers, practices and hospitals.’

*‘The lessons learnt section overlaps a lot with the discussion section.’*

At the start of the discussion, we summarize the main findings, which is common research practice. Subsequently, we discuss the findings in view of the literature and therefore have to repeat them briefly.

*‘Lines 117-126 – You cite a lot of literature on maternity care provider during COVID-19 but you don’t say how your study is adds to the existing literature – what is different in your study that has not already been looked at.’*

The previous studies were limited in their scope and only examined one aspect of providers’ experiences (for instance emotional health issues and experiences with e-Health). Our study has taken a more comprehensive view.

*‘Technically – qualitative samples cannot be representative. As far as I am concerned representative is more applied to quantitative survey – where the quantum of sample is representative of the larger category of those being sampled. That can never be the case. In qualitative studies its purposive and attempts to include all relevant categories in the sample – like has been done for this study.’*

What we originally meant was that we aimed to include respondents from different occupations in the field of maternity care, and also covering all geographical areas in the country. We have changed this sentence to: ‘We aimed to include a sample of all relevant categories of respondents, to cover […]’. We also changed the term ‘representative’ in other parts of the manuscript. (Line 158-159).

*‘I am not sure if the term ‘over sampled; is being used correctly in line 166-168. What the lines suggest to me that you included a higher sample of midwives and obstetricians because of the importance of their role. Oversampled as far as I know is used when you in your initial sampling you include a higher number than required so that even after non-response you would still get your required sample.’*

We changed this sentence to; ‘We purposefully included more community based midwives and obstetricians because they carry final responsibility for the care of women in their practice or hospital.’ (Line 163).
